# Calmodulin-Binding Transcription Factors: Roles in Plant Response to Abiotic Stresses

**DOI:** 10.3390/plants14040532

**Published:** 2025-02-10

**Authors:** Yayu Liu, Yali Qiao, Weibiao Liao

**Affiliations:** College of Horticulture, Gansu Agricultural University, Lanzhou 730070, China; liuyayu199809@163.com (Y.L.); 18409487294@163.com (Y.Q.)

**Keywords:** abiotic stress, CAMTAs/SRs, transcription factors, transcriptional regulation, stress responses, protein interactions

## Abstract

Plants face many abiotic stresses throughout their life cycle, such as drought, high temperature, low temperature, and salinity. To survive and reproduce, plants have evolved a complex and elaborate signal transduction network to sense stress signals and initiate corresponding defense mechanisms. Calcium ion (Ca^2+^), as a secondary messenger, plays an important role in mediating signal transduction in plant cells. Calmodulin (CaM) is an important class of Ca^2+^ receptors that sense changes in cellular calcium ion concentration and can interact with a range of proteins to regulate the activity of downstream target proteins. Calmodulin-binding transcription factors (CAMTAs) are a family of transcription factors (TFs) that are widely present in plants and can bind to CaM. The CAMTAs are regarded as the most characterized CaM-binding TF family in the plant Ca^2+^ signaling pathway. In recent years, studies have shown that CAMTAs play an important regulatory role in plant abiotic stress response and plant growth and development. Therefore, this review summarizes the recent progress in the discovery, structure, and role of CAMTAs under abiotic stresses, with a view to providing a reference for future CAMTA studies. Finally, the prospects and directions for further research on the potential mechanisms of CAMTAs in plants are also discussed.

## 1. Introduction

As an important nutrient and “secondary messenger” in plants, calcium ion (Ca^2+^) is widely involved in various cellular processes and plant growth and development, such as leaf senescence [1], seed germination [2,3], root growth [4,5], cell division, and photosynthesis [6]. Moreover, Ca^2+^ also participates in various biotic and abiotic stress responses, including diseases and insect pests, light, anoxia, osmotic stress, low- and high-temperature stress, and salt stress [7,8,9]. In addition, Ca^2+^ may maintain the stability of cell membranes and cell-wall structures [10,11]. The calcium signaling pathway has been considered to be one of the key signaling pathways in plants [12]. Plant responses to various abiotic stresses may be manifested as changes in intracellular free Ca^2+^ concentration [9].

To enrich the stimulus information and generate the corresponding cellular response, Ca^2+^ signals will be decoded and interpreted in the cell [13]. The realization of these processes mainly depends on calcium sensors and calcium-binding proteins [14]. Because these proteins have a high affinity for Ca^2+^, they can control the transmission of Ca^2+^ signals to a certain extent [14]. Studies have found that there are two types of calcium sensors in plants. The first type is called “sensor relays”, and its action mechanism is that conformational changes occur after binding to Ca^2+^, which in turn regulates the activity or function or gene expression of a variety of other target proteins, mainly including calmodulin (CaM), CaM-like proteins (CMLs), and calcineurin B-like proteins (CBLs). When the intracellular Ca^2+^ concentration changes, CaM can bind with Ca^2+^ to form the Ca^2+^/CaM complex through the EF-hand motif, thus changing the CaM conformation, and then regulating downstream target proteins to accomplish a series of cellular activities [14]. The binding of Ca^2+^ to CaM can be detected by techniques such as fluorescence resonance energy transfer (FRET) and genetically encoded calcium indicators (GECIs). Another calcium sensor has other reflection domains through which they pass signals to downstream targets. Such Ca^2+^ sensors are called “sensors responders”, including Ca^2+^-dependent protein kinases (CDPKs or CPKs), as well as Ca^2+^-and Ca^2+^/CaM dependent protein kinases (CCaMKs) [9]. Many transcription factors (TFs) have been considered calcium-binding proteins (CBPs), which are important mediators of Ca^2+^ to act as messengers. Many CBPs have been identified in plants, including calmodulin-binding transcription activators (CAMTAs)/signal responsives (SRs), MYB, WRKY, NAC, bZIP, CBP60, and MADS-box [15].

The CAMTA/SR family is one of the binding proteins of CaM, which contains CaM binding sites in its structural region [16]. After first discovered in *Nicotiana tabacum*, the CAMTA/SR family has been identified in various plant species [17,18,19,20,21]. A wide range of functions in plants are possessed by CAMTAs/SRs [17]. The CAMTAs/SRs have been shown to be involved in plant hormone signal transduction pathways [16]. Additionally, CAMTAs/SRs participate in abiotic and biotic stress responses, including diseases [22,23], insect pests [24], low temperature [25], salt [26], drought [27] and heavy metal stress [28]. To date, published reviews related to CAMTAs/SRs have mainly focused on their involvement in plant growth and development [29], stress responses, interactions with phytohormone signaling, or focused on a particular member of the CAMTA family. Despite the abundance of available literature on the function of CAMTAs/SRs, a comprehensive synthesis of recent findings is necessary to identify directions worthy of further research. In this review, we summarized the recent advances in how CAMTAs/SRs mediate plant adaptation to abiotic stresses. By integrating the recent findings, we aim to elucidate the action mechanism of CAMTAs/SRs interacting with other proteins in plant stress responses. We also highlighted the research progress on the transcriptional regulation of downstream genes by CAMTAs.

## 2. CAMTAs/SRs in Plants

### 2.1. Identification of CAMTA Gene Family Members in Plants

The tobacco *ethylene response 1* (*NtER1*) gene is the earliest *CAMTA* gene identified in plants, and it was named because it is highly homologous to the early tomato ethylene up-regulated gene, *ethylene-up-regulated 66* (*ER66*), and its expression is significantly induced by ethylene [30]. Subsequently, six homologs of *NtER1* were isolated in Arabidopsis (*Arabidopsis thaliana*), which were named *AtSR* or *AtCAMTA*, and the possible significance of this family member in many signaling pathways was indicated [30,31]. After that, *CAMTA* genes have been successively reported in several species, including *Avena sativa* [32], *Citrus sinensis* [33], *Vitis vinifera* [34], *Glycine max* [35], *Medicago truncatula* [36], *Phoebe bournei* [37], *Nicotiana tabacum* [38], *Phaseolus vulgaris* [39], *Sorghum bicolor* [40], *Rosa chinensis* Jacq. [21] and so on. Currently, 262 *CAMTA* genes in 42 plant species have been identified by genome-wide analysis (Table 1). With the advancement of bioinformatics analysis technology, Xiao et al. [25] screened 465 *CAMTA* genes from 112 plant genomes. They concluded that the *CAMTA* genes originated from the chlorophyta and gradually adapted to aquatic to terrestrial changes during the evolutionary process. This finding provided new insights into the molecular evolution of the *CAMTA* gene family.

### 2.2. Specific Motif Organization and Binding Sites of CAMTAs

CAMTA proteins have several conserved functional structural domains, including nuclear localization signal (NLS), CG-1 structural domain, transcription-associated immunoglobulin domain (TIG), ankyrin repeat domain (ANK), calmodulin-binding domain (CaMBD), and a variable number of IQ motifs [16].

The NLS can target proteins to the nucleus and thus function as transcription factors to activate transcription in target cells. Most NLSs are in the CG-1 structural domain at the N-terminus of CAMTA proteins, but there are exceptions. For example, two different types of nuclear localization sequences have been found for the rice (*Oryza sativa* L.) CAMTA protein (OsCBT), a dichotomous type located at the N-terminus and an SV40 type located at the C-terminus [20,64].

The CG-1 domain is an extremely conserved structural domain unique to eukaryotic multicellular organisms, consisting of approximately 130 amino acids. Through the CG-1 domain, CAMTA can directly initiate transcription by binding to DNA or indirectly by interacting with other transcription factors. In an earlier study, a part of the CG-1 DNA fragment was cloned from parsley, and its binding sites to target genes included the CGCG motif [65]. Subsequently, Yang et al. [66] found that Arabidopsis *AtCAMTA3*/*SR1* could bind the 6 bp (G/A/C) CGCG (T/G/C) sequence. Studies on Arabidopsis *AtCAMTA3* and rice *OsCBT* expanded the binding motif of CAMTA to CGbox [66]. This suggests that CAMTA/SR can regulate downstream genes by binding to specific regions of gene promoters.

The TIG structural domains are present in many functionally distinct transcription factors and can bind non-specifically to DNA and participate in protein dimerization [17]. However, the TIG is not essential for CAMTA proteins. Rahman et al. [40] found that some species of CAMTA/SR do not contain a TIG. ANK repeat sequences are repeat tandem modules of many eukaryotic proteins, viruses, and ANK repeat sequences can also be involved in protein–protein interactions [17]. The CaMBD is a conserved structural domain located at the C-terminus, consisting of 23 amino acids, and is the target site of CaM. After sensing the primary signal and delivering the signal to the cell, intracellular calcium levels increase, triggering CaM to bind CAMTA at the CaMBD structural domain. The IQ motif consists of a low-complexity region with the repeat motif IQXXXRGXXXX and is known to be associated with the binding of CaM and CaM-like proteins, and its binding to calmodulin can occur in both Ca^2+^-dependent and Ca^2+^-independent forms [29].

## 3. The Roles of CAMTAs in Abiotic Stress Responses

### 3.1. Salinity Stress

**Figure 1:**
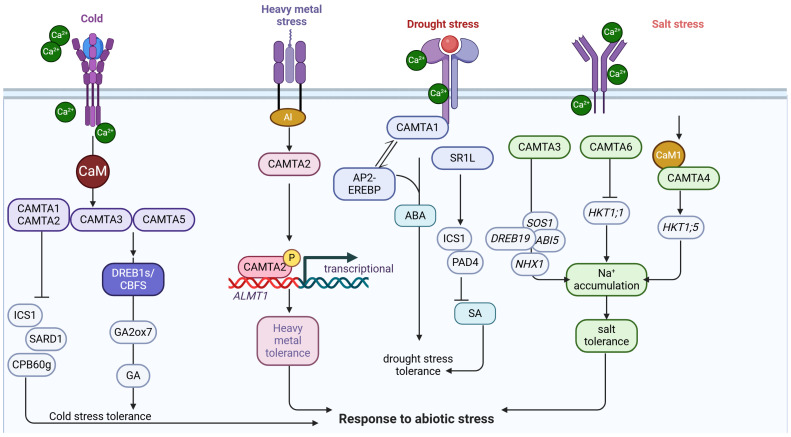
Overview of the mechanisms of the CAMTA response to abiotic stresses. CAMTA regulates gene expression, influences hormone homeostasis, maintains ionic homeostasis, and thus improves abiotic stress tolerance. Abbreviations: ABA, abscisic acid; SA, salicylic acid; GA, gibberellin.

In recent years, CAMTAs have been shown to play a key role in regulating the response to salt stress in a variety of plants [26,39,67,68]. Galon et al. [69] fused the GUS reporter gene into the *AtCAMTA1* promoter region of Arabidopsis and subjected the plants to different concentration gradients of salt stress, and histochemical analyses of the plants after 1 week revealed that the expression of GUS in the leaves increased with the increase in salt concentration. Through RNA-sequencing (RNA-seq) analysis, Prasad et al. [70] identified approximately 3000 genes regulated by AtCAMTA3/AtSR1 in Arabidopsis. Notably, chromatin immunoprecipitation–polymerase chain reaction (ChIP-PCR) results showed that AtCAMTA3/AtSR1 could bind the promoters of some salt-responsive genes, so they hypothesized that AtCAMTA3/AtSR1 could directly repress the expression of salt-responsive genes, thus negatively regulate salt tolerance in plants [70] (Figure 1). In addition to this, after RNA-seq sequencing of citrus root systems, Xie et al. [68] identified 1831 differentially expressed genes in citrus (*Citrus junos* Siebold cv.) that contained CAMTA transcription factors. It suggests that CAMTA is also involved in citrus response to salt stress, and this possibility and its mechanism deserve further investigation.

Shkolnik et al. [71] investigated the role of *AtCAMTA6* in Arabidopsis seed germination in response to salt stress. The authors found that *camta6* mutant seedlings could tolerate salt adversity up to 200 mM NaCl, and that Na^+^ uptake by germinating seeds was significantly reduced. Interestingly, the authors found that this phenomenon was related to the suppression of the expression of the *HIGHAFFINITY K^+^ TRANSPORTER1* (*AtHKT1;1*). The gene expression analysis showed that the expression of *AtHKT1* was restricted to the radicle and was not expressed in the cotyledons [71]. In contrast, the germination rate of *camta6 hkt1* double mutant seeds under salt stress was consistent with that of the wild-type (WT) plant, suggesting that *AtHKT1;1* is required for the insensitive character of *camta6* mutant seed germination to salt stress. Shkolnik et al. [71] analyzed transcriptome sequencing after salt stress in *Arabidopsis thaliana* and found that 62% and 84% of genes in WT seeds were under the direct and indirect control of *AtCAMTA6* during germination, respectively. The expression patterns of many salt stress-related genes (such as *SOS1*, *NHX1*, *ABI5*, and *DREB19*) were changed in *atcamta6*, and the promoter regions of many of these genes had an absolute dominance of the abscisic acid (ABA)-responsive element CACGTGTC [71] (Figure 1). It can be inferred that *AtCAMTA6* may regulate the response to salt stress during seed germination through the ABA signaling pathway, and may also coordinate the participation of downstream genes in the salt stress response by controlling other transcription factors.

Recently, Shen et al. [72] identified a novel calmodulin, HvCaM1, from the proteome study of barley (*Hordeum vulgare*) roots in response to salt stress. Phylogenetic tree analysis indicated that CaM1 originated from green algae and is a Ca^2+^-binding protein with a highly conserved sequence in plants. Furthermore, the expression of *HvCaM1* was higher in barley roots than in aboveground [72]. The *HvCaM1* knockdown (RNA interference, RNAi) lines showed higher salt tolerance than WT and overexpression (OE) lines under salt stress, which was attributed to the reduction in Na^+^ translocation rate from roots to aboveground in the RNAi lines [72]. The authors also showed the interaction between HvCaM1 and HvCAMTA4 by yeast two-hybrid assay (Y2H). Therefore, HvCAMTA4 may act as a target protein for HvCaM1 to differently regulate *HvHKT1;5* and *HvHKT1;1* expression, via binding to *cis*-regulatory elements in their promoters under salt stress [72]. The discovery that HvCaM1 and *HvHKT1;5* synergistically regulate salt tolerance in barley not only enriches the theory of crop salt tolerance, but also has an important guiding value for the genetic improvement of salt tolerance in barley as well as other cereal crops. This is consistent with the study of Huang et al. [65], who found that *HvHKT1;5* knockdown barley lines showed higher salt tolerance, with a significant reduction in Na^+^ translocation from roots to stems [73] (Figure 1).

Büyük et al. [39] investigated the expression of CAMTA under salt stress in resistant and sensitive varieties of cauliflower beans (*Phaseolus vulgaris*). Their quantitative reverse transcription-PCR (qRT-PCR) results showed that almost all CAMTAs were differentially expressed between the two varieties, and it was inferred that this group of genes plays a role in the stress response to salt stress. By analyzing *cis*-acting elements of *Cucurbita maxima* and *Cucurbita moschata*, Yuan et al. [46] found that most of the *CmoCAMTAs* and *CmaCAMTAs* were responsive to salt stress. Notably, transcriptional profiling of *CmoCAMTAs* and *CmaCAMTAs* under salt stress revealed similarities between *Cucurbita moschata* and *Cucurbita maxima* in terms of salt tolerance: the expression levels of *CAMTAs* were significantly induced in leaf vein but suppressed in leaf mesophyll in both plants [46] (Figure 1). The above studies have illuminated the significant roles of CAMTAs in plant salt stress responses. However, several intriguing questions remain. For instance, how do different CAMTAs precisely interact with each other and other signaling molecules in the complex salt stress-response network? Can the specific mechanisms by which AtCAMTA6 regulates salt stress response via the ABA signaling pathway be further dissected?

### 3.2. Heavy Metal Stress

In Arabidopsis, the root tip protected itself from aluminum (Al) toxicity by secreting malate through Al-activated AtALMT1 [74]. Through in vitro protein-DNA binding assays, it was found that AtCAMTA2 could activate *AtALMT1* expression under Al stress by binding to the *ACGCGT* in the *AtALMT1* promoter [74]. Therefore, the authors suggest that CAMTA2 could play a positive regulatory role in heavy metal stress tolerance by directly activating the expression of *AtALMT1* [74] (Figure 1). Kakar et al. [38] identified 29 *CAMTA* genes in four tobacco species. Notably, four *NtabCAMTA* genes were significantly up-regulated in leaves and seven genes were significantly up-regulated in roots after cadmium (Cd) treatment [38] (Figure 1). Therefore, we can speculate that genes up-regulated in leaves may be involved in regulating processes related to photosynthesis protection and scavenging of reactive oxygen species (ROS). In roots, the up-regulated genes might be involved in regulating the uptake of cadmium ions by root cells to reduce the transport of cadmium ions to the above-ground parts. A recent study has unveiled that tomato (*Solanum lycopersicum* L.) *CAMTA3*/*SR1* exhibited a robust response to a spectrum of abiotic stresses, especially Cd stress [19]. This discovery underscores the pivotal role of *SlCAMTA3*/*SR1* in orchestrating plant defense mechanisms against environmental adversities, highlighting its potential to enhance stress tolerance in crops [19]. This may imply that *SlCAMTA3*/*SR1* may be at the position of a node in a signal transduction network that is capable of integrating multiple abiotic stress signals and thus coordinating plant defense responses. However, studies on CAMTA’s response to heavy metal stress are limited. Therefore, there is great potential for research in this area. Moreover, the functional roles of CAMTAs in non-model plants, particularly crops, remain largely unexplored.

### 3.3. Extreme Temperature as a Stress Factor Including Heat and Cold Stress

*CRT*/*DRE Binding Factor* (*CBF*)-mediated signaling pathways play an important role in the regulatory pathway of cold stress response. The CBF family can directly bind to the promoter regions of some cold-responsive genes and regulate the expression of these genes. The CBF family genes identified so far are *CBF1*, *CBF2*, and *CBF3*, also known as *DREB1b*, *DREB1c*, and *DREB1a* [75]. Doherty et al. [76] showed that Arabidopsis CAMTA3 could bind to the CM2 (CCGCGT) motif in the *CBF2* promoter region and positively regulate *CBF2* expression. However, the difference between the cold tolerance of the *camta3* mutant and that of the WT was not obvious, and the expression patterns of *CAMTA1* and *CAMTA3* were relatively similar at low temperatures. The *camta1 camta3* double mutant was further obtained, and the cold tolerance of the double mutant was weakened after 7 d of cold domestication, which suggests that *CAMTA1* and *CAMTA3* may be jointly involved in the regulation of cold tolerance in Arabidopsis thaliana [76]. Furthermore, Lee and Seo [77] demonstrated that Arabidopsis CAMTA3 interacted with HHP2, and the expression of *CBF2* in the *hhp2-1* mutant after cold induction was like that in the *camta3* mutant [76]. This suggests that HHP can participate in the transcriptional regulation of CBF together with CAMTA3. Kidokoro et al. [78] found that Arabidopsis CAMTA3 and CAMTA5 could induce the expression of *CBF1*/*DREB1b* and *CBF2*/*DREB1c* during a sudden drop in temperature, but these two genes were not involved in the response to a slow decrease in temperature.

Du et al. [79] found that there was no significant difference in the growth status of the Arabidopsis *camta3*/*sr1* mutant and the wild type at 25 °C, but the growth of the *camta3*/*sr1* mutant was significantly inhibited at 20 °C, and a significant increase in salicylic acid (SA) content was also found, which suggests that *camta3*/*sr1* is very sensitive to temperature drop. Like this idea, Kim et al. [80] examined the SA content in the *camta1 camta2 camta3* triple mutant, *camta1 camta2*, *camta1 camta3*, and *camta2 camta3* at 22 °C, and found that the SA content of the triple mutant was significantly increased, and that expression of *ICS1*, *CBP60g*, and *SARD1*, genes related to SA accumulation, was also significantly increased in the triple mutant [80]. The expression of *ICS1*, *CBP60g,* and *SARD1*, which are genes related to SA accumulation, was also significantly increased in the triple mutant and *camta3* mutant, which indicated that there was functional redundancy among CAMTA1, CAMTA2, and CAMTA3, and all of them could inhibit SA synthesis. Only CAMTA3 was induced after 4 °C treatments, which might explain why the *camta3* mutant was sensitive to the decrease in te79]. To further clarify how CAMTA3 represses SA pathway gene expression at low temperatures, Kim et al. [81] transformed the *CAMTA3* promoter-regulated CAMTA3-GFP mutant protein into *camta2 camta3* double mutant plants and found that the expression of *CAMTA3-GFP* repressed the expression of SA pathway genes in the double mutant [81]. While previously Du et al. [79] suggested that the inhibition of SA pathway gene expression by *CAMTA3* required CaM binding to CaMBD. Kim et al. [81] found that CAMTA3^334^, which did not contain CaMBD, had a more pronounced effect on SA pathway gene expression and inhibited SA biosynthesis. Inconsistent with this view, Chao et al. [82] suggested that the temperature regulation of CAMTA3 gene induction activity was independent of the C-terminal CaMBD. The results suggest that the CG-1 domain of CAMTA3 confers the ability to induce gene expression by the CAMTA3 transcription activation domain (TAD).

In addition, CAMTA can be involved in responding to cold stress through alternative splicing. Wei et al. [56] analyzed the variable shear patterns of CAMTA in two poplar trees, *Populus trichocarpa* and *Populus ussuriensis*, under cold stress. Seven shear variants, *PtCAMTA1*~*PtCAMTA7*, were obtained. Kim et al. [83] localized two loci, *qSCT1* and *qSCT11*, associated with cold resistance in rice by quantitative trait locus (QTL), and CAMTA is one of the candidate genes for qSCT1, which might be helpful for the breeding of cold-tolerant varieties of rice. The qRT-PCR analysis revealed that most of the *PtCAMTA* splice variants were down-regulated in roots of *Populus trichocarpa* under cold stress, and most of the genes were up-regulated in leaves within a short period of time due to cold-induced expression [83]. In addition, the different expression patterns of CAMTA in the leaves of *Populus trichocarpa* and *Populus ussuriensis* may explain the differences in cold tolerance between them (Figure 1). In short, CAMTA may be involved in cold stress response by inducing the expression of CBF family genes and inhibiting SA synthesis. Additionally, CAMTAs/SRs in many species also respond to ABA. However, it is not known whether there is an ABA-dependent signaling pathway associated with CAMTAs/SRs at low temperatures.

In addition to cold stress, high-temperature stress is also one of the extreme environmental factors affecting plant growth and development. However, not many studies have been conducted on the involvement of CAMTA in plant response to high-temperature stress. Kan et al. [84] found that the heat-tolerant gene *TT2* could affect the change of Ca^2+^ concentration, and CaM sensed the changes in Ca^2+^ concentration to repress the expression of the CAMTA homologous gene *SCT1*, which in turn regulated the deposition of the waxy layer, leading to enhanced heat tolerance in rice [84]. Moreover, CAMTA expression levels were significantly induced in several species under high-temperature conditions. For example, *PbCAMTA6*, *PbCAMTA1*2, and *PbCAMTA8* were significantly expressed at high temperatures in *Phoebe bournei* [37], and the CAMTA family also showed a significantly strong response to high-temperature stress in wheat (*Triticum aestivum* L.) [61]. In contrast, SlCAMTA3/SR1 in tomatoes may not be sensitive to heat stress [19]. However, more studies are needed to confirm the exact involvement of CAMTA genes in plant response mechanisms to heat stress.

### 3.4. Drought Stress

Previous research evidence suggests that the drought response is primarily driven by biosynthesis and signaling of the phytohormone ABA. Plants use root-to-aboveground signaling to adapt to drought conditions of varying intensity and duration [85]. It is also the case that CAMTAs play an important role in plant response to drought stress. Compared with wild-type plants, root development in *camta1* mutants was abolished under drought stress and the mutants showed a higher sensitivity to drought and reduced survivability, indicating that AtCAMTA1 probably was involved in drought stress response [86]. Microarray analysis further revealed that *AtCAMTA1* regulated drought stress by activating ABA signaling. In addition, AP2-EREBP transcription factors were also the key regulatory components of AtCAMA1 in response to drought stress [86]. AP2-EREBP was previously shown to modulate plant response to drought stress [76]. Thus, CAMTA1 interacted with ABA and AP2-EREBP in response to drought stress.

Silencing of *SlSR1L* resulted in decreased drought stress tolerance in tomatoes, which accelerated water loss in leaves [77]. Furthermore, the expression of *SlSR1L* was significantly induced in detached leaves and whole plants by drought stress [87]. Thus, *SlSR1L* is a positive regulator in plant drought stress response. Compared with wild-type plants, root development in *camta1* mutants (silencing of the *AtCAMTA1* gene) was abolished under drought stress and the mutants showed a higher sensitivity to drought and reduced survivability, indicating that AtCAMTA1 probably was involved in drought stress response [86]. Recently, a study by Zeng et al. [27] knocked out the *SR1* gene and overexpressed the *SR1* gene. The *sr1* mutant was found to be more sensitive to drought stress and had a higher rate of water loss than the WT. In addition, the *sr1* mutant showed reduced response to ABA in seed germination, root elongation, and stomatal closure [27]. They also found that the drought-sensitive and ABA-insensitive phenotypes of the *sr1* mutant were restored by knockout of the SA synthesizer ICS1 or the activator PAD4. Several drought/ABA-responsive genes showed differential expression in *sr1* mutant and *sr1* overexpressing plants [27]. These results suggest that SR1 plays a positive role in drought stress tolerance, whereas SR1 negatively regulates SA accumulation and thus antagonizes the drought response.

In *Phaseolus vulgaris*, different levels of drought stress reduced the number of flower buds and nodes with a related reduction in yield [88]. Furthermore, severe drought stress reduced this trait more than moderate drought stress. Relative expression of the gene indicated that moderate drought induced the expression of *PhavuCAMTA1* gene. On the other hand, the level of *PhavuCAMTA1* decreased under severe drought stress. These results indicated that the expression of *PhavuCAMTA1* increased at moderate drought stress to overcome drought stress.

*GmCAMTA12* is known to be involved in regulating the expression of network-related regulatory genes, especially with the CGCG/CGTTG motif under drought stress in Arabidopsis and soybean (*Glycine max*) hairy roots [35,89]. The transgenic Arabidopsis and chimeric soybean (overexpressing *GmCAMTA12*) plants exhibited enhanced drought survivability and performed better growth and development under drought stress compared with the non-transgenic counterparts [35]. *GmCAMTA12* overexpression improved the drought survival efficiency, germination efficiency, and root length of Arabidopsis and improved the regeneration of more developed and drought-efficient hairy roots in soybean [35]. In contrast to this study, Baek et al. [89] analyzed the role of the soybean *CAMTA* family in response to abiotic stress. They found that overexpression of *GmCAMTA2* and *GmCAMTA8* was found to cause Arabidopsis to be unusually sensitive to drought, with accelerated water loss and down-regulation of stress-responsive genes compared with wild-type plants. These findings suggest that *GmCAMTA2* and *GmCAMTA8* might be negatively associated with drought stress tolerance and regulated by circadian rhythms [89]. They may serve as potential targets for improving crop stress tolerance. Therefore, GmCAMTA2 and GmCAMTA8 appear to negatively regulate drought tolerance, while GmCAMTA12 seems to have a positive role, making it a promising candidate for improving plant resilience to drought. These studies provide valuable insights into the complex role of *CAMTA* genes in plant abiotic stress responses, highlighting the potential for genetic engineering to enhance crop survival under water-limited conditions (Figure 1). In addition, recent studies have shown that the CAMTA family is involved in the response to drought stress in several species, such as *Musa acuminata* [55], *Camellia sinensis* [33], *Heimia myrtifolia* [51], and *Phoebe bournei* [37]. Although the specific molecular mechanism of CAMTA in response to drought stress remains to be determined, these data provide new directions for future research. Therefore, we concluded that the enhancement of plant drought tolerance by CAMTA is closely related to the activation of the antioxidant system, the robustness of the osmoregulatory system, and the control of hormonal signaling pathways.

### 3.5. General Stress Responses

General stress response (GSR) is a series of rapid basic responses that are universally evolved conservatively in different species when various environments are favorable [90]. GSR could regulate the part of stress response genes and was partially modulated by the *rapid stress response element* (*RSRE*) [91]. Some studies show that CAMTA3 plays a positive role in plant GSR [92,93,94]. *RSRE* was highly rich in some early stress-response promoters, including wounding, UVB, or osmotic stress, flagellin 22 (flg22), oligogalacturonic acid (OGA), cold, osmotic and UVB stress-induced genes, which suggested that *RSRE* might be a functional GSR in the rapid stress-triggered genes [93]. Fla22 and OGA with Ca^2+^ burst-induced capability could strongly trigger the expression of the luciferase gene downstream of the *RSRE*. *RSRE::LUC* activity in wounded and H_2_O-treated plants was significantly higher than that in unwounded and calcium chelator ethylene glycol tetraacetic acid (EGTA)-treated plants, indicating that Ca^2+^ might have an important role in wounded-promoted *RSRE* activity [93]. The activity of *RESE::LUC* together with *35S::CAMAT3* in leaves was higher than the activity of *RSRE::LUC* or *mRSRE::LUC* alone, indicating that CAMTA3 could trigger *RSRE* activity and activate the expression of downstream reporter genes through *RSRE*. *RSRE*-mediated injury response was synergistically promoted by CAMTA2, CAMTA3 and CAMTA4 [93]. Hence, *RSER* could regulate the GSR by activating the expression of CAMTA gene family members. The *camta3* mutant showed a decreased *RSRE* activation [94]. The activity of *4×RSRE: LUC* in *camta3-4 mekk1-5* double mutant was similar to that in *camta3* mutant, suggesting that CAMTA3 might be the downstream of MEKK1. MEKK1 loss-of-function *MITOGEN-ACTIVATED PROTEIN KINASE (MAPK) KINASE KINASE1* (*mekk1-5*) mutant showed programmed cell death (PCD) and had a high level of hydrogen peroxide (H_2_O_2_), ROS and SA, which was the symbol of hypersensitivity response (HR), indicating that HR may play a role in the *RSRE* phenotype of *mekk1-5* mutant [94]. Furthermore, the basal and constitutive levels of *RSRE* in *camta3* and *constitutively expressing hydroperoxide lyase1* (*ceh1*)/*camta3* were greatly reduced, confirming that CAMTA3 is the main *RSRE* transcriptional activator. By comparing the SA levels in *ceh1*, *camta3,* and *che1*/*camat3* mutants, CAMTA3 was used as a positive regulator of SA levels in *ceh1* mutants [92]. *RSRE* was specific in conferring methylerythritol cyclodiphosphate (MEcPP)-mediated reactions, and CAMTA3 played a key role in MEcPP-mediated RSRE induction [92]. The expressions of GSR model genes, *CRK14* and *WRKY48*, and the *RSRE*-containing UPR genes, *inositol-requiring protein-1* (*IRE1a*) and *basic leucine zipper 60* (*bZIP60*), were significantly decreased in *ceh1*/*camta3* mutant than in *camta3* mutant [92], indicating that CAMTA3 might be an important transcriptional activator of RSRE to trigger some stress-response genes. Consequently, MEcPP as a key dynamic metabolic effector mediated rapid stress response by CAMTA3. By comparing the transcriptome data of Arabidopsis under seven molecular model treatments at different times in the early stage, Bjornson et al. [95] found that genes up-regulated within 10 min of treatment were enriched with many CAMTA transcription factor binding elements. The CAMTA family binding core element CGCG is a major transcriptional regulator of GSR in plants [95]. These findings further confirm the involvement of CAMTA in GSR and reveal the commonality and specificity of transcriptional events in the early immune response in plants and identify new important components of the plant immune response. Recently, Zeng et al. [96] revealed how the plastid metabolite MEcPP activated the stress-response regulatory center of RSRE. They identified the HATI/TPL/IMPa9 inhibitory complex in which HAT1 binds directly to the RSRE and its activator CAMTA3, shielding the RSRE and isolating the activator. Activation of MEcPP after stress induction disrupted the complex, exposing RSRE and releasing CAMTA3, and MEcPP also promoted Ca^2+^ inward flow and increased nuclear Ca^2+^ levels, which are critical for CAMTA3 activation and initiation of RSRE gene transcription [96]. Thus, these results provide new insights into the relationship between CAMTA and RSRE. Therefore, future studies can further delve into the specific regulatory mechanism of CAMTA in GSR, including its interaction with other transcription factors and signaling pathways. The in-depth study of the relationship between CAMTA and GSR is expected to provide theoretical support for the cultivation of more resistant crop varieties.

## 4. Molecular Mechanisms of Calcium Signaling in Response to Abiotic Stress Through CAMTA

### 4.1. CAMTA Interacts with Other Proteins in Response to Abiotic Stresses

Liu et al. [97] observed that the calcium-dependent protein kinase CPK5 caused protein conformational instability by phosphorylating serine at position 964 of CAMTA3 in Arabidopsis, thereby attenuating the transcriptional repression of downstream resistance-related genes. This interaction realized rapid signaling from stress signal perception to gene transcriptional regulation, which ensured that plants could respond to the threat of abiotic damage in a timely manner. Wang et al. [98] identified AtCAMTA5 as a possible reciprocal protein of BZR1 when analyzing key components of the BR signaling pathway in Arabidopsis thaliana. AtCAMTA5 could regulate the expression of *CBF2*, which is a target gene of BZR1 and BZR2 [98,99], suggesting that AtCAMTA5 may be involved in the BR-mediated signaling pathway. Zhang et al. [100] showed that amino acids 282–498 of GCN5 interacted with the ANK structural domain of CAMTA2, which in turn regulated histone acetylation, leading to smaller grains and reduced starch. However, it is not clear how they synergized to regulate the accumulation of storage proteins [100]. Studies are needed to validate the molecular mechanism by which GCN5 and CAMTA2 synergistically regulate storage protein accumulation.

### 4.2. CAMTA-Mediated Transcriptional Regulation of Downstream Target Genes

**Figure 2:**
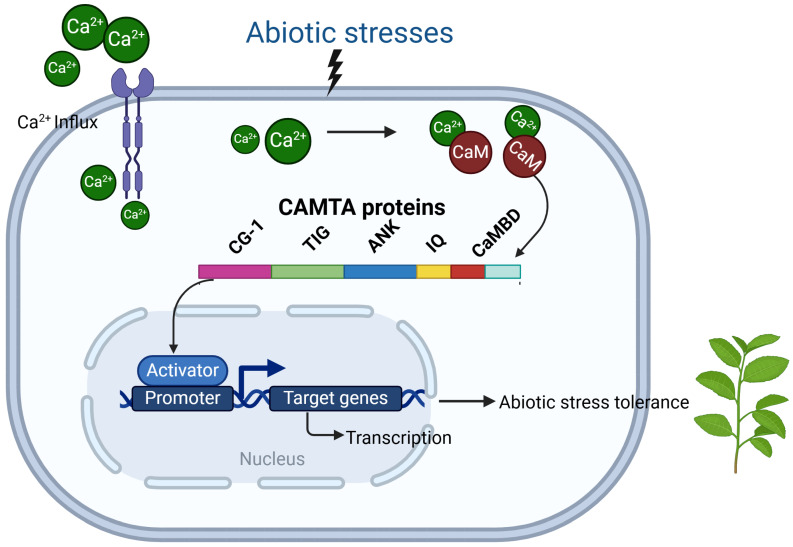
The proposed model for CAMTA-mediated signaling in plants.

CAMTA transcription factors can directly bind to the promoter regions of downstream genes through the CG structural domain and regulate the transcription of downstream genes (Figure 2). The binding of CAMTA to the promoter regions is sequence-specific, and it can interact with other transcription factors and co-activators to enhance or repress gene expression. For example, CAMTA3 regulated *CBF2* expression by binding to the CM1/2 sequence in the promoter region of *CBF2*. The expression of *CBF2* and several downstream *COR* genes was repressed in *camta3* mutants, and the *camta3-camta1* double mutant had a cold-sensitive phenotype [76]. Interestingly, computational analysis identified 30 genes that were significantly induced by cold stress, and hypergeometric testing revealed that CG-1 elements were enriched in genes rapidly induced by low temperature [76]. This indicates that CG-1 sequences are important for the early cold response of the AtCAMTA protein. In addition, Kakar et al. [38] found that tobacco genes (*NbenCAMTA1* and *NbenCAMTA2*) that were significantly induced under cold stress contained two CG-1 structural domains. Therefore, focusing on whether the CG-1 structural domains are working properly might help plants to better tolerate the cold. EIN3 is a very important transcription factor in the ethylene signaling response pathway. Both in vivo and in vitro binding assays have confirmed the binding of SR1 to the promoter region of *EIN3*. The expression of the *EIN3* gene was elevated in the *sr1* mutant but reduced in *sr1-4D*, suggesting that SR1 negatively regulated *EIN3* expression [101]. Rahman et al. [40] found that the expression of some relevant genes of the defense signaling pathway was significantly elevated in *camta3*/*sr1* mutants subjected to biotic stress. Electrophoretic mobility shift assay (EMSA) and chromatin immuno-precipitation (ChIP) assays also further demonstrated that AtCAMTA3/SR1 interacted with the CGCG-box in the promoter region of target genes to play a role in plant growth. Similarly, Yuan et al. [102] showed that AtSR1 acted as a transcription factor in plant growth and immune homeostasis by interacting with the CG-box-containing “CGCG” in the promoters of its target genes [102]. In addition, *AtCAMTA1* triggered the expression of the GUS reporter driven by the P281 promoter of *AVP1* but did not stimulate the expression of the GUS reporter in the mutant in which the CGCG-box was disrupted, suggesting that *AtCAMTA1* attached to the CGCG- box enhances *AVP1* expression [103]. In addition, CAMTA3 plays a role in cold-induced expression of *CBF1* and *ZAT12*. These findings provide a potential link between calcium and cold-regulated gene expression. Sun et al. [104] also demonstrated by ChIP assays that *CBP60g* was a direct target gene of AtCAMTA3/SR1, and that autoimmunity to *atcamta3*/*sr1* was suppressed by the *sard1 cbp60g* double mutant and the *N*-hydroxypipecolic acid (NHP) synthesis-deficient mutants *ald1* and *fmo1* [104]. These results suggest that the utilization of CGCG motifs by CAMTA varies among plant species. In Arabidopsis, several CAMTA family members have been shown to specifically recognize CGCG motifs on different genes and synergistically regulate various stages of plant growth and development. In crops such as rice, it was found that CAMTA also utilized similar CGCG motifs to regulate the *Sus2* and *SBEIc* genes, which in turn affected the genes responsible for grain size and grain weight in wheat, providing potential targets for crop genetic improvement [100].

## 5. Conclusions and Prospective

Until now, 262 CAMTA proteins have been identified in 42 plant species. In this review, we summarize recent advances in the CAMTAs family in response to abiotic stresses and their mechanisms of action. We illustrate how CAMTAs function as members of calcium signaling. In addition, although significant progress has been made in the role of CAMTA in a variety of environmental stresses such as drought, heavy metal toxicity, cold, and salt stress, there are still many questions that need to be answered. For example, many target genes that may be regulated by CAMTA/SR have been identified by sequencing, but further studies on the specific regulatory mechanisms of CAMTA/SR in different environments are still needed to better define the role of CAMTA/SR. Furthermore, the crosstalk between CAMTA and other transcription factors and signaling pathways in plant adversity response remains to be elucidated. Finally, CAMTA/SR members in many species are responsive to ABA although the *cis*-acting element that binds to CAMTA/SR is similar to the ABA response element. However, there is no direct evidence that CAMTA/SR is involved in ABA signaling. Future studies should focus on these aspects in order to gain a more comprehensive understanding of the role of CAMTA transcription factors in plant stress response and to provide a theoretical basis for the enhancement of plant stress tolerance through genetic engineering. In the future, we can improve the species through gene editing and other technological means to obtain excellent disease-resistant and resilient varieties.

## Figures and Tables

**Table 1:** CAMTA/SR members identified by genome-wide analysis.

Species	Number	References
*Arabidopsis thaliana*	6	[31]
*Avena sativa*	20	[32]
*Brachypodium distachyon*	7	[41]
*Brassica napus* L.	18	[40]
*Brassica campestris ssp. chinensis* Makino	8	[42]
*Camellia sinensis*	6	[33]
*Chenopodium quinoa*	7	[43]
*Citrus sinensis*	9	[44]
*Cucumis sativus* L.	4	[18]
*Cucurbita maxima*	6	[45]
*Cucurbita moschata*	5	[45]
*Capsicum annuum*	5	[46]
*Durio zibethinus*	10	[47]
*Eleusine coracana*	7	[48]
*Fragaria vesca*	3	[49]
*Heimia myrtifolia*	10	[50]
*Liriodendron chinense*	2	[51]
*Malus domestica*	8	[49]
*Manihot esculenta*	6	[22]
*Mangifera indica*	8	[52]
*Medicago truncatula*	7	[36]
*Medicago sativa*	17	[53]
*Musa acuminata*	5	[54]
*Glycine max*	15	[35]
*Nicotiana tabacum*	13	[38]
*Nicotiana benthamiana*	5	[38]
*Nicotiana sylvestris*	6	[38]
*Nicotiana tomentosiformis*	5	[38]
*Oryza sativa* L.	7	[20]
*Phaseolus vulgaris*	8	[39]
*Phoebe bournei*	17	[37]
*Phyllostachys edulis*	11	[55]
*Populus trichocarpa*	7	[56]
*Prunus persica L. Batsch*	5	[57]
*Pyrus communis*	9	[49]
*Pyrus bretschneideri*	9	[49]
*Prunus avium*	4	[49]
*Prunus mume*	4	[49]
*Rubus occidentalis*	4	[49]
*Rosa chinensis* Jacq.	5	[21]
*Sesamum indicum* L.	5	[58]
*Solanum lycopersicum*	7	[59]
*Solanum melongena*	28	[46]
*Sorghum bicolor*	6	[60]
*Triticum aestivum* L.	17	[61]
*Vigna radiata*	8	[62]
*Vitis vinifera*	10	[34]
*Zea mays* L.	9	[63]

## Abbreviations

The following abbreviations are used in this manuscript:

Ca^2+^	Calcium ion	CaM	Calmodulin
CAMTAs	Calmodulin-binding transcription factors	TF	Transcription factor
CMLs	CaM-like proteins	CBLs	Calcineurin B-like proteins
FRET	Fluorescence resonance energy transfer	GECIs	Genetically encoded calcium indicators
CDPKs	Ca^2+^-dependent protein kinases	CCaMKs	Ca^2+^-and Ca^2+^/CaM dependent protein kinases
CBPs	Calcium-binding proteins	SRs	Signal responsives
NLS	Nuclear localization signal	TIG	Transcription-associated immunoglobulin domain
ANK	Ankyrin repeat domain	CaMBD	Calmodulin-binding domain
RNA-seq	RNA-sequencing	ChIP-PCR	Chromatin immunoprecipitation–polymerase chain reaction
WT	wild type	ABA	Abscisic acid
RNAi	RNA interference	OE	Overexpression
Y2H	yeast two-hybrid assay	qRT-PCR	Quantitative reverse transcription-PCR
Al	Aluminum	Cd	Cadmium
SA	Salicylic acid	TAD	Transcription activation domain
QTL	Quantitative trait locus	GSR	General stress response
RSRE	Rapid stress-response element	OGA	Oligogalacturonic acid
EGTA	Ethylene glycol tetraacetic acid	PCD	Programmed cell death
H_2_O_2_	Hydrogen peroxide	ROS	Reactive oxygen species
HR	Hypersensitivity response	EMSA	Electrophoretic mobility shift assay
